# Characteristics of adolescent boys who have displayed harmful sexual behaviour (HSB) against children of younger or equal age

**DOI:** 10.1186/s40359-020-00490-6

**Published:** 2020-11-19

**Authors:** Monica Jensen, Sanne C. Smid, Tormod Bøe

**Affiliations:** 1 Betanien Hospital, V27, Regional Clinical Resource Team Working With Children and Adolescents Who Have Displayed Problematic and Harmful Sexual Behavior, Bergen, Norway; 2 Department of Methodology and Statistics, Utrecht University, Utrecht, The Netherlands; 3 Department of Psychosocial Science, Faculty of Psychology, University of Bergen, Bergen, Norway

## Abstract

**Background:**

Adolescents who have displayed harmful sexual behaviour (HSB) are often described as a heterogeneous population, but different offender-, offense-, or victim-based typologies have been proposed. Two common typologies are based on the victim’s age and/or on offender–victim age discrepancies.

**Methods:**

Using data from a Norwegian clinical sample, we aimed to investigate the characteristics of two subgroups of adolescents: those with younger/child victims (HSB-C) and peer victims (HSB-P). The sample was collected from a public child and adolescent psychiatry outpatient clinic. *N* = 54 boys, mean age 14.1 (younger age: HSB-C, *n* = 30, equal age: HSB-P, *n* = 24). Based on patient records, all patient registries within the sample were reviewed retrospectively. The data were analysed in R with different statistical tests (e.g. N − 1 chi-square test, Fisher’s exact test).

**Results:**

After adjusting the *p* values for multiple comparisons, none of the test statistics showed significant results. Based on the magnitude of the effect-size estimates, the following tendencies and potential meaningful differences emerged: more adolescents in the HSB-C group had experienced their own trauma early (*V* = 0.42), had more than one victim (*V* = 0.32), and had displayed repeated HSB (*V* = 0.27), and their caregivers required extensive interventions (*V* = 0.20). More adolescents in the HSB-P group had cognitive abilities in the normal/high range (*V* = 0.32), and their caregivers more often had difficulties acknowledging the need for support and treatment (*V* = 0.20).

**Conclusions:**

The nonsignificant differences between the subgroups, despite some strong and moderate effects, bring about a discussion on the utility of using “victim age” in combination with the “offender–victim age differences” as the sole classification criterion for adolescents who have displayed HSB. The heterogeneity of the adolescent HSB population and lack of reliable information on more homogenous subgroups dynamics will remain challenges for clinicians and other practitioners needing a broad assessment and intervention focus.

## Background

Estimates suggest that 30–50% of child sexual abuse (CSA) involves other children or adolescents who had displayed harmful sexual behaviour (HSB) [1, 2]. The UK definition of HSB, adopted here, will be “one or more children engaging in sexual discussions or acts that are inappropriate for their age or stage of development. These can range from using sexually explicit words and phrases, inappropriate touching, using sexual violence or threats to full penetrative sex with other children and adults” [3].

Adolescents who have displayed HSB are often described as a heterogeneous population with diverse aetiologies and pathways into HSB [4–6]. Different offender-, offense-, or victim-based typologies have been proposed to improve our response to and understanding of factors that contribute (or not) to the aetiology of problematic and HSB in more specific HSB subgroups. Identification of more distinct HSB subgroups regarding, for instance, motivational and etiological factors would be desirable for the adolescents, their families, and the practitioners. Subgroups that are more distinct will, for example, be expected to improve and given more accurate, timesaving, and narrower risk and need assessments [4, 7]. The alternative would leave the practitioners to deal with the full heterogeneity within the HSB population and would require broader and more time-consuming assessment methods to evaluate and inform each adolescent’s individual safety and intervention plan.

One of the ways to explain and understand HSB among adolescents is by looking at the age group of their victims [4, 7]. Common typologies are based on the victim’s age and/or on offender–victim age discrepancies [8, 9]. HSB-Children (HSB-C) will further be defined as the group of adolescents with child victims below age 12 who are at least 4 years younger. HSB-Peer (HSB-P) will be the group of adolescents with equal-age victims, up to 4 or fewer years younger/older. The literature also describes subgroups of adolescents with both peer/adult victims (HSB-P/A) and mixed-aged victims (HSB-M). Although these groups are interesting [9–11], we will not discuss them further because the adolescents in these subgroups were few in number or /were outliers and thereby were excluded from our study.

The offender–victim age distinction has proven to be meaningful for adult offenders (“rapists” versus “child sexual abusers” [12, 13]), but has produced results that are more variable in studies of HSB adolescents [7, 11]. However, the categorizations for adolescents based on the victim’s age and/or offender–victim age distinction has produced some promising consistent findings across studies [7, 9, 12, 14–18]. In their critical review of the HSB literature, Keelan and Fremouw [11] listed some common methodological limitations of victim-age based studies: inconsistent definitions, low-powered studies, lack of standardized measures, and recidivism data based solely on conviction rates. Prior studies mainly are based on samples from residential or correctional settings, so the typology’s utility has been investigated to a lesser degree among younger people participating in outpatient treatment [7, 11].

To summarize some of the relevant findings on differences linked to our study, adolescents in the HSB-P group a more likely to use force or violence, have indicators of delinquency, have criminal records and arrest histories [10, 14, 15, 17–19], and offend against female strangers [9, 10]. Adolescents in the HSB-C group have fewer behavioural problems, have more internalizing and social problems [14–17], more often have a history of sexual abuse [18], and offend against both family members or acquaintances of both sexes [7, 9, 14].

The aim of the present study was to investigate factors related to typologies based on victim age and/or offender–victim age discrepancies (HSB-C versus HSB-P), drawing on experiences from practice with a Norwegian clinical outpatient sample of adolescent boys. Very few empirical studies have been conducted on problematic and harmful sexual behaviour in Norway, so the study could be important for further development of our understanding and response to these HSB subgroups. It is also important to generate more knowledge and utility regarding adolescent HSB- and victim age-based subgroups.

Based on the more consistent findings apparent in previous summarized literature, we hypothesized that the HSB-C group in our Norwegian clinical sample (1) experienced more traumas, especially sexual abuse; (2) were more often closely related to their victims; and (3) more often had both males/females as victims, in comparison to the HSB-P group. On the other hand, the HSB-P group was expected to (4) have more antisocial tendencies; (5) have more acquaintances and strangers as victims; and (6) have mostly female victims. Finally, we wanted to compare HSB-C and HSB-P offenders regarding their social, family, and cognitive functioning; self-esteem; and emotional loneliness. However, due to missing replications or inconsistencies in previous literature, this will be more inductive and experience based because we are not in a position to pose specific hypotheses concerning the expected pattern of results for these variables.

### The Norwegian context

Norway has a population of about 5.4 million. About 21% are children under the age of majority (18 years). The age of sexual consent is 16 years, and the age of criminal responsibility is 15 years. Around 4% of all minors (< 18 years) get help or constraint decisions from the Child Welfare Service (CWS) regarding maltreatment and/or serious behavioural problems (e.g. HSB). About 60% of the group of minors who are clients of the CWS live with their families, while 40% reside in outplacement settings (74% in foster care, 18% in housing with follow-up, and only 8% in residential care) [20].

Until recent years, Norway has lacked a systematic and differentiated multiagency assessment and intervention for children and adolescents who have displayed problematic or harmful sexual behaviours [21, 22]. The HSB interventions for adolescents have historically been unsystematic, with mixed risks, and mainly treatment oriented, delivered through public health and social outpatient services (e.g., by the CWS or the Children & Adolescent Mental Health System [CAMHS]). Within the juridical and correctional system, in addition to the police, the Barnahus [23] and the Mediation Service [24] were established in later years as important collaborators for the CWS and CAMHS, including for HSB cases. In general, Norway only has two prison units for adolescents (aged 15–18 years) with room for 8–10 total prisoners). Far from all cases with serious behavioural problems are adjudicated or registered at the police/Barnahus level, but most children/adolescents with serious behavioural problems will be registered with Child Welfare Services (CWS). The public structure and orientation of services in Norway illustrate the politicians’, government’s, and practitioners’ historical commitment to and preference for mainly developmental and treatment-oriented help/services for minors and their families [22].

To differentiate the severity of sexual behaviours (online/offline) and use it correspondingly, in recent years [25], multi-disciplinary professionals in Norway have used a translated version of the original Australian “Traffic Light” [26] in combination with an adapted definition continuum from the UK [1]: “Ok/healthy sexual behaviour (green light)—Problematic sexual behaviour (yellow light)—Harmful sexual behaviour (red light)”.

## Methods

### The total sample and the HSB-C and HSB-P subgroups

A convenience sample was collected from an HSB outpatient clinical unit associated with children and adolescent psychiatry in western Norway. The referrals there mainly originate from outpatient clinics within the child/adolescent mental health system (CAMHS/BUP) and municipal CWS. The aim of the original study was to examine the characteristics of minor boys in Norway who had committed HSBs against other minors (aged 0–18 years) [27]. The data were collected from closed patient records. From the total sample of 66 cases, 10 were excluded (girls and cases that were rejected or not started), resulting in a final sample of 56 boys referred to the HSB unit between 2004 and 2013. Their mean age at first contact with the unit was 14.1 years (*SD* = 2.1). Jensen et al. [27] found that the majority of the boys lived with their biological families (89%) and that they were mostly executing the HSB in their own homes or in the vicinity (76%). Additionally, 76% of their victims were girls. The majority (59%) of the victims (mean age 8.5 years) were primary school aged (6–11 years), while 19% were preschool aged (0–5 years) at the alleged time of the assault. The majority of the boys who had displayed HSB were related to their victims as neighbours/friends (41%), siblings (34%), or other relatives (16%). The HSBs were primarily “hands-on” behaviour—kissing/touching the breasts/genitals (27%) and penetrative sexual acts (64%). More than half of the boys (62%) had displayed two or more HSBs prior to referral.

**Fig. 1:**
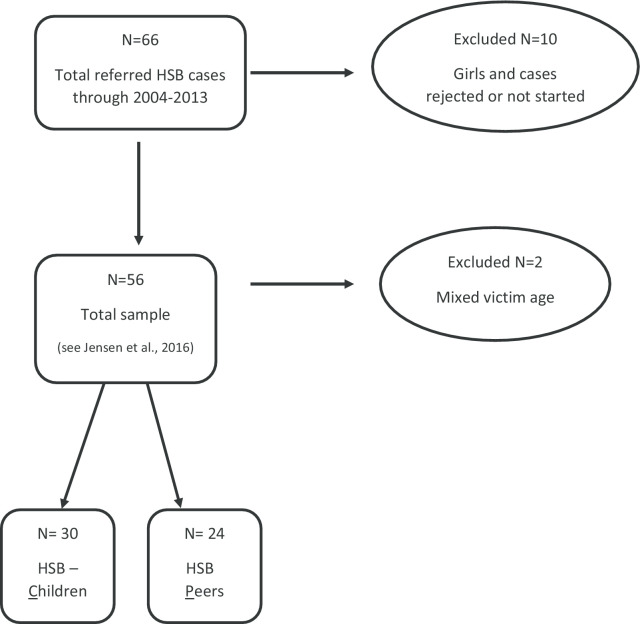
Flowchart depicting sample selection

In present study, adolescent boys who had committed HSB against children who were younger than 12 years old (c.f., victim age) *and* 4 or more years younger than themselves (c.f., offender–victim age differences) were classified into the HSB-C group (*n* = 30). The HSB-P group consists of adolescents who had committed HSBs against victims less than 4 years younger or older than themselves (*n* = 24). The categorization scheme used (4 years younger or older) was based on prior studies (e.g., [9]) and Norwegian practice definitions. The adult- and mixed-aged-targeting group (*n* = 2) was excluded from further data analysis (Fig. 1).

### Procedure and ethics

Based on the HSB agency’s patient records, one specialist in clinical psychology who had been working in this area for more than 25 years retrospectively reviewed all of the patient registries within the sample. The data were treated according to the guidelines set by the Norwegian Data Protection Official for handling of confidential and sensitive information (cf. The Norwegian Health Personnel Act 26). The data collection was approved by the Norwegian Centre for Research Data as a quality-assurance project, and it was not deemed obligatory to notify the regional committee for medical and health research ethics.

The possible inherent subjectivity from the single coder summarizing the written sources in the patient records must be noted. The professional coded the data for the purposes of a quality-assurance project, before anyone had thought of or established hypotheses for this study. As a validity assessment, the coding of certain variables (e.g. “repeated HSB”) was reassessed by two other experienced team colleagues (both specialists in clinical psychology with more than 25 years of experience), without deviations from the primary coder’s results. Unfortunately, the practitioners did not implement psychometric inter-rater checks in this initial process, and the patient registries are no longer available for such an analysis.

### Measurements

#### Care situation at disclosure

The care situation at disclosure was coded from static data in the HSB unit’s/CAMHS’ electronic patient records. The response choices were (1) living with biological parents (including living with two biological/adoptive parents) or with one biological/adoptive parent alone or with one stepparent; or (2) living with foster parents or with caregivers in a child welfare institution.

#### Carer qualities

The coding information for carer qualities was drawn from several written sources in the patient records—specifically, from the CWS’ documents/evaluations, evaluations from multidisciplinary case meetings, and/or the HSB unit’s/CAMHS’s final evaluation report. The response choices were (1) the caregivers require extensive therapy/interventions, (2) the caregivers/family are the main helpers, and (3) the caregivers/family have difficulties acknowledging their need for support/treatment.

For assessment, this variable was operationalized and coded based on and adapted from Hall’s [26] subdivision of parental/caregivers’ participation and support, and how this can affect the HSB treatment’s effects in different ways. Response (1) includes the parents/caregivers/families who could have extensive need for broad help, more than just help/treatment to prevent the further occurrence of HSB. Response (2) includes the parents/caregivers/families who were evaluated as their adolescent’s primary and most valuable helpers, and response (3) includes parents/caregivers who could have problems accepting the accusation related to HSB. They could have problems engaging and supporting the adolescent’s/family’s need for HSB treatment/interventions.

#### Sexual behaviour

The coding information for sexual behaviour was collected from the patient records, interviews with the adolescents who have admitted to HSB, referral information, victim-based information collected from the police/Barnahus, or the final HSB unit/child and adolescent mental health service (CAMHS) evaluation. The response choices were (1) “sexual intercourse” (intercourse, intercourse-like, mouth-to-genital contact, forced masturbation), (2) “sexual actions” (kissing, touching of genitals), and (3) “other sexual behaviour” (sexualized behaviour such as flashing, masturbation in front of others, showing pornography, and other technology-assisted HSBs). These categorizations follow Norwegian legislation.

#### Location of HSB

The coding for location of HSB was drawn from referral information about the referral HSB, victim-based information from the police/Barnahus, and/or interviews with the adolescent who admitted to performing HSB. The response choices were (1) “in the offender’s home or the vicinity”, (2) “in the victim’s home or the vicinity”, (3)”at childcare/school/after-school care”, and (4) “other private/public place”. Here, “in the offender’s home or the vicinity” (1) includes parent home(s) but also foster or residential homes/placements and the vicinity. It also includes cases in which the adolescent who committed HSB and the victim live in the same home. “Other private/public place” (4) includes, e.g., HSB displayed on public transport or at cinemas, swimming pools and parties in friends’ homes.

#### Repeated HSB

Repeated HSB was coded based on information in the patient records collected from interviews with the adolescent/parents/caregivers, school reports, multi-agency case meetings, and/or the final HSB unit/CAMHS’s evaluation report. This variable includes registered HSB events that took place between the commencement and termination of outpatient treatment contact at the HSB unit. The response choices are “yes” or “no”.

#### Other criminal/norm-breaking behaviour

Other criminal/norm-breaking behaviour was summarized and coded from several written sources in the patient records (e.g., from formal assessment, information from the police/Barnahus, the CWS’s evaluation/documents, and summaries of interviews with the adolescent and parents done by the executive case therapist). The response choices were yes or no; “yes” includes reported serious non-sexual norm-breaking behaviour problems such as considerable school skipping, shoplifting/stealing, severe lying or non-obedience to adults, vandalism, violence, drug/alcohol use, arson, and/or pet and animal abuse (cf. World Health [28] F91 diagnosis). The response “no” includes cases in which the patient records explicit report “no serious non-sexual behavioural problems” and cases in which no information or focus on serious behavioural problems has been registered (“not known or not current”).

#### Own traumas

The variable “own trauma” was coded from several sources in the patient record,—especially interviews with the child and parents/caregivers, information/evaluation from CWS or police/Barnahus, or the final HSB unit/CAMHS’s evaluation. The response choices were (1) “no”, (2) “assault”, (3) “multiple traumas”, (4) “neglect”, and (5) “concern for trauma”. “No” was coded when the patient record contained an assessment and evaluation of potential exposed trauma/abuse but no disclosure thereof. “Assault” was registered when the adolescents who committed HSB had experienced one kind of assault (physical or sexual assault or bullying). The assault could have been a single occurrence or repeated assaults but of the same kind. “Multiple traumas” was coded when one experienced a combination of two or more types of assault or neglect (multi-traumatized; [29]). “Neglect” was coded when one experienced general considerable maltreatment and/or emotional neglect [30] (c.f. interventions from CWS). “Concern for trauma” was coded when the executive practitioners reported clinical concern that the child had experienced assault, but no further trauma evaluation/assessment was documented in the patient’s record to confirm or set aside this hypothesis. We collected no data on witnessing violence/abuse.

#### Social functioning

The coding information for social functioning was drawn from assessment measures completed by the child, parents, or school (the ASEBA instruments; [29], and the Development and Well-being Assessment; [30]), from multidisciplinary case meetings and/or the HSB unit’s/CAMHS’s final evaluation report.

The response choices were (1) “concerning functioning”, which includes adolescents having friends but those who are often superficial, concerning, and antisocial acquaintances, including cases in which the adults have difficulties with obtaining an overview of the adolescent’s social contacts and activities; (2) “isolated function”, which includes adolescents who have few or no friends in school and their spare time and who mostly spend time alone or together with younger children; and (3) “good functioning”, which includes adolescents having close prosocial friends, with whom they partake in after-school activities and have registered no serious concerns.

#### Cognitive abilities

Cognitive abilities was coded when cognitive abilities had been measured with at least one standardised test, due to the patient records’ timing, mostly with the Wechsler Intelligence Scale for Children (WISC-3 [*n* = 29] or WISC-IV [*n* = 11]) [31–34]). Due to Norwegian practice, the response choices were 1) “normal/high abilities” for IQ 85 or above or 2) “general/specific learning difficulties” for IQ 70–85. Here, specific learning difficulty is in the same IQ interval as general learning difficulties (70–85) but in combination with ICD-10, F80-81 diagnosis [35]. Additionally, response (3) was “moderate/mild intellectual disability” (cf., “learning disability”), for IQ 35–69.

#### Self-esteem and emotional loneliness

Self-esteem and emotional loneliness were measured with the Self-Esteem (SE) and Emotional Loneliness (EL) Scales from the Adolescent Sexual Abuser Project (ASAP) [36, 37]. Previous evaluations have suggested that these scales have adequate psychometric properties [37, 38]. The normal ranges of the scales (ASAP-SE = 4–7; ASAP-EL = 27–45) are based on a sample of 92 non-offending British adolescent males [36].

### Statistical analysis

The data were analysed using R (version 3.4.4; [39]). Chi-square tests were used to investigate the association between the 15 categorical outcome variables and the HSB categories. Based on the expected frequencies of the cells in the contingency tables, it was decided whether the *N* − 1 chi-square test or Fisher’s exact test would be used, as recommended by Campbell [40]. Three variables had expected frequencies below 1—age at own trauma, sex of the victim, and location of HSB—and were therefore analysed using Fisher’s exact test. All of the other categorical variables had expected frequencies of 1 or higher and were analysed using the *N* − 1 chi-square test as highly recommended by Campbell [40], using R code based on Busing et al. [41]. For all of the categorical variables, the effect size measure Cramer’s *V* was computed because this effect-size measure is suitable for 2 × 2 and larger contingency tables [42].^1^

The non-parametric Wilcoxon rank-sum test was used for the three continuous variables, as the data were not normally distributed [43]. A corresponding effect size measure *r* was computed for these three variables.

Finally, to control for multiple comparisons, the *p* values of the 18 tests were adjusted by using the Holm correction in the *stats* package of R [44]. This correction strongly controls the overall alpha level but is less conservative than the Bonferroni correction is [45].

In the current study, 9.47% of the values were missing. To follow standard procedures, we checked whether there were associations in the data between missingness and the HSB categories. See Table 2 for an overview of the variables, which contain missing values, and the results of these association tests. Without correcting *p* values for multiple comparisons, associations between missingness and HSB categories were found for the variables of caregiver qualities and repeated HSB. These associations disappeared after correcting for multiple testing. Furthermore, there was no reason to suspect systematic differences in the missingness between the two HSB categories (see Table 1) because the missing values were mainly caused by the time span of the data collection and changing clinical practices over time. Note that the study used data collected over a 10-year range. The earliest data included some instruments that were not used later, and vice versa. Importantly, the participants were categorized into HSB-C and HSB-P by the researchers of the current study and not by the clinicians who actually saw the participants. Because of the study design, there was no reason to expect missingness to be related to the HSB categories, and any association was assumed to be purely incidental. For more details on all of the analyses performed and the annotated R code, we refer to our extensive analysis report available here: https://goo.gl/LQXmc2.

**Table 1:** Descriptive information stratified by HSB-category

	N (% missing)	HSB-C *N* = *30*	HSP-P *N* = *24*	Combined *N* = *54*	Test statistic
Ethnicity	54				χ_1_^2^ = 1.3, *p* = 0.38^1^
Born in Norway		87%	96%	91%	
Born outside of Norway		13%	4%	9%	
Care situation at disclosure	54				χ_1_^2^ = 1.4, *p* = 0.28^a^
Biological/adoptive parents		80%	92%	85%	
Foster parents/institution		20%	8%	15%	
Carer qualities	50 (7.4)				χ_2_^2^ = 3.9, *p* = 0.16^a^
Caregivers require extensive therapy/interventions		63%	40%	54%	
Caregivers/family are main helpers		27%	30%	28%	
Caregivers/family have difficulties acknowledging support/treatment		10%	30%	18%	
Sexual behaviour	54				χ_2_^2^ = 0.67, *p* = 0.79^a^
Sexual intercourse		67%	58%	63%	
Sexual actions		27%	29%	28%	
Other sexual behaviour		7%	12%	9%	
Debut age HSB, *M* (*SD*)	54	14.6 (1.6)	13.4 (2.5)	14.1 (2.1)	*F*_1 52_ = 2.7, *p* = 0.11^b^
Number of HSB events	54				χ_2_^2^ = 1.8, *p* = 0.43^a^
3 or more		60%	50%	56%	
1		30%	46%	37%	
2		10%	4%	7%	
Location of HSB	53 (1.9)				χ_3_^2^ = 2.8, *p* = 0.52^a^
Offender’s home or vicinity		80%	78%	79%	
Victim’s home or vicinity		13%	4%	9%	
Childcare/school/after-school care		3%	13%	8%	
Other private/Public place		3%	4%	4%	
Number of HSB victims	54				χ_2_^2^ = 11, *p* < 0.05^a^
1		40%	83%	59%	
2		47%	8%	30%	
3 or more		13%	8%	11%	
Sex of victim	54				χ_2_^2^ = 3.3, *p* = 0.24^a^
Female		63%	83%	72%	
Male		30%	17%	24%	
Male and female (mixed)		7%	0%	4%	
Offender-victim relation	54				χ_4_^2^ = 4.8, *p* = 0.32^a^
Neighbour/friend		37%	50%	43%	
Biological-/half siblings		23%	33%	28%	
Other relative		20%	12%	17%	
Foster-/step siblings		13%	0%	7%	
Stranger		7%	4%	6%	
Repeated HSB	50 (7.4)				χ_1_^2^ = 3.8, *p* = 0.064^a^
Yes		27%	5%	18%	
No		73%	95%	82%	
Other criminal/norm breaking behaviour	46 (14.8)				χ_1_^2^ = 0.02, *p* = 1^a^
Yes		70%	68%	70%	
No		30%	32%	30%	
Own traumas	43 (20.4)				χ_4_^2^ = 10, *p* < 0.05^a^
No (not known)		29%	47%	37%	
Assault		12%	37%	23%	
Multiple traumas		21%	5%	14%	
Neglect		25%	0%	14%	
Concern for trauma		12%	11%	12%	
Age at own trauma	41 (24.1)				χ_3_^2^ = 8.2, *p* < 0.05^a^
0–5		43%	6%	27%	
6–11		9%	28%	17%	
12–15		4%	6%	5%	
Not applicable		43%	61%	51%	
Social functioning	45 (16.7)				χ_2_^2^ = 2.5, *p* = 0.31^a^
Concerning		44%	44%	44%	
Isolated		44%	28%	38%	
Good		11%	28%	18%	
Cognitive abilities	40 (25.9)				χ_2_^2^ = 8.6, *p* < 0.0
Normal/ high abilities (IQ = 85 or greater)		26%	71%	45%	
General/specific learning difficulties (IQ = 70–85)		61%	18%	42%	
Moderate/mild intellectual disability (IQ = 35–69)		13%	12%	12%	
Self-esteem, *M* (*SD*)	41 (24.1)	6.3 (2.2)	6.1 (1.6)	6.2 (1.9)	*F*_1 39_ = 0.95, *p* = 0.34^b^
Emotional loneliness, *M* (*SD*)	40 (25.9)	35.3 (8.0)	39.6 (9.5)	36.9 (8.7)	*F*_1 38_ = 1.2, *p* = 0.27^b^

N is the number of non-missing values

Tests used: ^a^Pearson *X*^2^ with simulated *p* values; ^b^Wilcoxon test

## Results

Most adolescents in our sample were born in Norway (91%) and lived with at least one of their biological/adoptive parents (85%). For more than half of the sample, the adolescent’s parents required extensive therapy/interventions, and around 20% of the parents had difficulties acknowledging the need for support/treatment. Slightly less than 50% of the adolescents had concerning social functioning. Most of the adolescents in the sample also engaged in other criminal or norm-breaking behaviours (see Table 1).

Table 1 shows descriptive information stratified by HSB category, combined with the results of the statistical analysis. After adjusting the *p* values for multiple comparisons, none of the test statistics showed significant results. However, when looking at the effect sizes of *V* and *r*, we can conclude the following.^2^ Large effects were found for age of own trauma (*V* = 0.42), with the majority of those experiencing trauma early belonging to the HSB-C group; cognitive abilities (*V* = 0.32), with the majority of adolescents with normal/high abilities belonging to the HSB-P group; and number of HSB victims (*V* = 0.32), with the majority of adolescents who had more than one victim belonging to the HSB-C group.

A medium effect was reported for repeated HSB (*V* = 0.27), with most of the adolescents who repeated HSBs during the treatment period belonging to the HSB-C category. The results also show medium effects for caregiver qualities (*V* = 0.20), suggesting that more caregivers for adolescents in the HSB-C group required extensive interventions, while more caregivers for adolescents in the HSB-P group have had difficulties acknowledging the need for support and treatment. For all other variables, small effect sizes and effect sizes between small and medium were reported. Only for other criminal/norm-breaking behaviour was no clear association found (*V* = 0.02), suggesting adolescents in both groups are just as likely to display these kinds of behaviours.

## Discussion

The aim of the current study was to investigate factors related to offender, victim, and HSB characteristics in a clinical outpatient sample of Norwegian adolescent boys who were categorized as belonging to either the HSB-C or the HSB-P group. Following adjustment for multiple testing, the main finding was that no statistically significant differences existed between the adolescents in the two groups. However, the magnitude of some of the effect size estimates point to potentially meaningful group differences. Therefore, it is reasonable to assume that the non-significance could have been caused by a lack of power because of the small sample size. These potential meaningful group differences should be investigated further in a larger clinical outpatient sample. We want to comment some of the potential group differences here.

Both groups had largely experienced traumas, which supports prior research regarding exposure to trauma as one risk factor in developing problematic or harmful sexual behaviours [18, 30, 46]. Note that we coded the exposed assaults reported in the patient records. We did not code if, how, or with whom adolescents have shown or been assessed/evaluated for trauma symptoms. Contradictory to our expectations, there were no significant differences between the groups (c.f. Hypothesis 1). This could be due to our small sample. However, we found a large effect size regarding the age of the offender’s own trauma, in that the boys in the HSB-C group experienced trauma in their first 5 years more often, whereas those in the HSB-P group had later exposure, most often between 6 and 11 years of age. It is hard to say what this means because we did not collect information about examples to provide the exact frequency and duration of their own traumatic experiences, detailed information about how they were traumatized, or who their offenders were (age, sex, and relationship). A theoretical hypothesis is that exposure to prior trauma motivates the display of HSB by an offender identifying and repeating their own trauma on victims whose ages correspond to the offender’s own age when traumatized [47, 48]. Especially for the HSB-C group, we know that an early onset of trauma in general could have a negative impact on further development and functioning (e.g., neurodevelopmental, trust, attachment and relational, intimate and sexual, emotional and behavioural; [46]).

Second, we found a tendency towards a large effect size regarding cognitive abilities. More adolescents in the HSB-P group had IQ scores in the normal/high range (> 85 IQ), whereas those in the HSB-C more often had lower IQ scores and functioning (70–85 IQ), possibly related to the experience of early trauma [46]. Lower cognitive functioning for the boys in the HSB-C group could have implications for their difficulties in social relations with peers and for identification with younger children [18]. This inclination might also restrain them from building more age-adequate intimate and sexual relations with peers [10]. The lower cognitive functioning in the HSB-C group could also imply less sophistication and regulation related to their HSB (c.f. more repeated HSBs that are easier to disclose). Importantly, concerning discrepancies in victim age and cognitive function, we did not collect or compare data on the adolescents’ physical strengths or degree of social status (c.f. use of power in different ways related to abuse).

In the present study, we could not distinguish the boys in the HSB-C group who in fact had a specific sexual need/drive towards younger children from those motivated by other developmental, emotional, or situational factors [49]. However, a theoretical hypothesis is that physical maturation and sexual development in puberty, together with situational factors such as victim access and a lower IQ, facilitate HSB against younger children—such as in a kind of a developmentally appropriate socio-psychological relationship [10]. This view of the HSB-C group would be qualitatively different from understanding and approaching them as adolescent boys motivated by “atypical sexual interest” in young children.

Third, the results show a large effect size regarding the number of HSB victims. Those in the HSB-C group more often offended against multiple victims (two or more), whereas most of the HSB-P group members had one victim. This tendency could support the importance of situational factors such as victim access—with the younger victims being perhaps more vulnerable to and less protective of and resistant to adolescents’ HSB. This also indicates a theoretical hypothesis that the HSB-P boys were displaying HSB in relation to one known peer victim (i.e., more sophisticated and relationally motivated) but the HSB-C boys were displaying HSB to different victims (i.e., less sophisticated and more situationally motivated).

We found a moderate effect size regarding the HSB-C group repeating more HSBs during treatment contact. This could be interpreted as an indicator of the treatment effect but could also be related to other factors, including when during the assessment/treatment period the new repeated HSB was registered, the kind of new HSB, caregiver abilities and awareness, and how the boys were able to conceal their new HSB [27]. These other factors were unavailable from the journal information, so it could not be accounted for in the results.

In summarizing our effect size results, which suggest that the HSB-C boys experienced traumas earlier, had lower cognitive function, had more victims, and had displayed more repeating HSBs, we could support a pathway model to HSB that contains basic attachment and social skills deficits in interactions with peers. They might have less discretion in victim choice, be less sophisticated, have more cognitive/emotional/social identification with younger children, and have more access to this group of potential victims [4, 7–10, 50]. Additionally, we clearly need to know more beyond the HSB-C boys’ individual characteristics such as their caregivers’ background, qualities, supervision, and monitoring (e.g., [51]).

For the HSB-P group, the tendency of moderate effect size for less HSB repetition and lower number of victims as well as the number of prior disclosed HSBs not differing between the groups could imply that the boys in the HSB-P group more often repeated HSBs with selected peer victims. Clinically, it might be interesting to assess more extensively whether this represents a more specific problem for these HSB-P boys with regulating their sexual behaviour and/or a lack of situational comprehension of consent in an age-appropriate intimate relationship. This could also apply to the HSB-C boys, but then this might represent a more general and expected problem due to, for example, their lower cognitive function and the possible impacts of experiencing trauma at a younger age.

We found a moderate effect size indicating that the HSB-C caregivers required more extensive help than the HSB-P caregivers did. Being a caregiver for an adolescent who has been strongly suspected of or who has disclosed having displayed HSB against other minors was, in itself, a shocking and extreme life event in both groups [51–53]. For the caregivers in the HSB-C group, having an adolescent who had displayed HSB against much younger children can provoke strong emotions that include shame, guilt, social isolation, and fear for their child’s development and future. This reaction might also affect the caregivers’ own motivation and involvement, thus influencing the interpretation, assessment, and evaluation process regarding the HSB-C caregivers’ need for therapy and intervention [51, 54].

As discussed earlier, the strong effect for the HSB-C boys of experiencing their own trauma at a young age and the moderate effect of caregivers’ need for extensive help might also indicate insufficient or absent age-adequate care, supervision, and monitoring in general before the disclosure of HSB. This finding might also imply a theoretical hypothesis of more family-related and attachment problems, resulting in challenges with regulating intimate relations in general for the boys in the HSB-C group [12, 50].

Relatedly, we found a moderate effect pertaining to HSB-P parents having more difficulties acknowledging and supporting the recommended therapy/interventions. When the victim is the same age as the child, the caregivers might see the HSB as more of a sexual-exploration event in a mutual, age-appropriate intimate relationship. Therefore, they may exhibit less acknowledgement of, and support for, the professionally recommended HSB-related therapy/intervention—both for their adolescent and for themselves as a family.

A surprising finding was the lack of differences/effects between the subgroups with regard to social functioning or the presence of other criminal and norm-breaking behaviour (c.f. Hypothesis 4). Based on the literature and previous research [12, 13, 15, 18], we would have expected that the HSB-P group members would report more criminal and norm-breaking behaviour. Our study does not support this, but as previously mentioned, this could be due to our sample size. Both groups included mostly adolescents with concerning or isolated social functioning. A majority in both groups had non-sexual behaviour problems in addition to HSB. This could be a result of the Norwegian outpatient sample being less selective and including more adolescents with both sexual and non-sexual harmful behaviour (i.e., “generalists”; 4, 13, 18, 22).

Furthermore, based on earlier research, we expected to observe differences between the HSB-C and HSB-P groups for offender–victim relations and victim gender (c.f., Hypotheses 2, 3, 5, and 6). The pattern of results did not provide strong indications for such group differences, suggesting that victim characteristics were not key distinguishing factors between the groups. These findings instead could be understood in terms of the situational pathway to HSB for both groups (i.e., access to victims; [4, 18, 55].

Finally, the mean scores of self-esteem (the ASAP–SE) and emotional loneliness (ASAP–EL) for adolescents in both groups were, slightly surprisingly, within the “normal” range [37, 38]. These results suggest that the ASAP did not adequately discriminate between different subgroups of adolescents who have displayed HSB or between the adolescents in the sample and non-clinical samples of adolescents. These results question the ASAP’s validity in assessing these factors, and/or the results show no support for the clinical assumption that a low self-esteem score and a high emotional loneliness score are important psychological offender-specific characteristics, as has been previously suggested [14, 36].

## Limitation and future research

There are several limitations to the present data material. We have collected data from a natural clinical sample; therefore, the missing data vary by variable. This is expected, considering the data are based on patient records that were registered for clinical use by different professionals and practitioners over time. One should also be aware of the possibly inherent subjectivity of using a single coder in analysing the patient record information. Furthermore, the patient record data were registered over a 10-year period during which the professional knowledge, agencies, terms, definitions, identification, understanding, and methodologies related to the HSB group have undergone continuous development and change (e.g. the development and understanding of technology-assisted HSB; [56]).

Some researchers have commented that the lack of differences or not many differences between HSB subgroups could refer to the natural variation in the nature of adolescent behaviour [9, 37] or that contextual and situational factors have greater impacts on youth behaviour than backgrounds and personality factors do [18]. In research on adults, it is well accepted that there are clear differences between “child sexual abusers” and “rapists” [12, 13], but the results based on victims’ age are far more inconsistent for the adolescents who commit HSB [7, 9, 12, 55].

Alternative typologies other than victim age, which have been proposed as useful for further exploration, could include victim gender combined with victim age, offense characteristics (e.g., frequencies, violence, penetration, online, and/or offline), relationship to victim (siblings/close relations vs acquaintances/strangers), sexual maturation (cf. “Tanner scale”), or delinquency and criminal history [57–59].

A crucial limitation of the present study is the small sample size [11]. Given the small sample size, the power probably is low. Therefore, failing to detect any significant differences between the HSB categories was somewhat expected, especially because a strong correction to control for multiple comparisons results in a loss of power. Furthermore, we did detect large and medium effects; thus, it is reasonable to assume that the nonsignificance could be caused by a lack of power because of the small sample size. Another issue related to the small sample size is generalizability, which is frequently questionable when the sample size is small. On the other hand, the target group of this study historically is naturally low frequency and difficult to access in outpatient clinics (CAMHS).

The subtyping of HSB has generally provided inconsistent findings across studies. To move this field further, one approach may be to conduct systematic, clinical, single-case studies that bridge knowledge from clinical experience and research and through longitudinal studies into longer-term outcomes for adolescents belonging in, for example, either the HSB-C or the HSB-P group. It may also be beneficial to capitalize on multicentre collaborations with pooled samples to enable high-powered investigations of group characteristics using robust statistical methods. It would also be worthwhile to develop international guidelines for research and move towards common definitions and operationalizations in this area.

## Conclusion

The nonsignificant differences between the subgroups, despite some strong and moderate effects, question the utility of using victim age, in combination with offender–victim age differences, as the sole classification criterion for adolescents who have displayed HSB. Clearly, based on the small sample in our study, we cannot conclude anything but can just point out some potential meaningful differences between the groups. In the immediate future, the heterogeneity of the adolescent HSB population will remain a challenge for clinicians and other practitioners. As long as clinicians continue to lack reliable information on more homogenous subgroups regarding, for instance, dynamic risk factors and strengths, they must ensure a broad assessment and intervention focus.

Additionally, we will emphasize the need for more continuous and ecological evaluations of HSB-oriented treatment in clinical day-to-day work (during/after therapy sessions) and over more long-term durations (more single-case and longitudinal studies). More evaluations of therapies/interventions and safety plans will inform the adolescents and their carers to better prevent and reduce/stop repeating HSB, but also to further develop practitioners’ understanding, methods, and skills for helping the adolescents and their families in differentiated ways.

Finally, the results from the present study suggest a developmental understanding of why some adolescents have displayed HSB against younger children. We suggest that practitioners should be cautious in further fortifying labels such as “perverse” and “abnormal” according to, for example, adolescents’ sexual drive and attractions. Rather, we encourage a greater understanding of these boys, in light of their resemblance to their younger victims in terms of cognitive, linguistic, sexual, and social functioning. This perspective should not be used as an excuse to explain why adolescents have displayed HSB against younger children. However, it should help practitioners to better understand these adolescents’ possible natural identification and contact with younger, more vulnerable victims and assist them with moving on in a more prosocial and prosexual developmental direction [49]. In contrast, if an initially broad assessment shows an adolescent who has displayed HSB toward younger children and has a combination of *good* cognitive, social, and/or family functioning and no known trauma, this may suggest a need for more extensive assessment and for treatment of possibly abnormal sexual attraction and drive towards younger children.

## Data Availability

https://goo.gl/LQXmc2. The analyses are available. Data are available from Betanien Hospital, but restrictions apply to the availability of these data, which were used under license for the current study and thus are not publicly available.

## Appendix

See Table 2.

**Table 2:** Descriptive information and association test results of missing values

Variable	Missing values in HSB-C	Missing values in HSB-P	Total number missing values	Test statistic and *P* values before Holm correction	*P* values after Holm correction
Social functioning	3	6	9	χ_(1)_^2^ = 2.12, *p* = .145^*N*^	*p* = 1.00
Other criminal/ norm breaking behavior	3	5	8	χ_(1)_^2^ = 1.22, *p* = .270^*N*^	*p* = 1.00
Self-esteem	5	8	13	χ_(1)_^2^ = 1.20, *p* = .156^*N*^	*p* = 1.00
Emotional loneliness	5	9	14	χ_(1)_^2^ = 2.96, *p* = .086^*N*^	*p* = .684
Own trauma	6	5	11	χ_(1)_^2^ = 0.01, *p* = .940^*N*^	*p* = 1.00
Age own trauma	7	6	13	χ_(1)_^2^ = 1.13, *p* = .287^*N*^	*p* = 1.00
Cognitive abilities	7	7	14	χ_(1)_^2^ = 0.23, *p* = .630^*N*^	*p* = 1.00
Location HSB	0	1	1	df = 3, *p* = 0.4^F^	*p* = .100
Caregiver qualities	0	4	4	χ_(1)_^2^ = 5.30, p = .021^*N*^	*p* = .213
Repeated HSB	0	4	4	χ_(1)_^2^ = 5.30, p = .021^*N*^	*p* = .213

Only variables with missing values are shown in the table, all other variables did not contain missing values. Two tests are performed: N − 1 Chi-Square test, and Fisher’s exact test, denoted by ^N^ and ^F^ respectively. Note that all *p* values became > .213 after adjustment by using holm correction

## Abbreviations

CSA: Child sexual abuse
HSB: Harmful sexual behaviour
HSB-P: Harmful sexual behaviour against peers
HSB-P/A: Harmful sexual behaviour against peers/adults
HSB-C: Harmful sexual behaviour against younger children
HSB-M: Harmful sexual behaviour against a combination of peers, adults, and/or younger children (mixed victim age)
CWS: Child Welfare Services (maltreatment and serious behaviour problems)
CAMHS: Children & Adolescent Mental Health Services (in Norway: BUP/BUPA)
IQ: Intellectual quotient
ASAP: Adolescent Sexual Abuser Project
SE: Self-Esteem Scale (ASAP)
EL: Emotional Loneliness Scale (ASAP)

## Footnotes

1 When Fisher’s exact test is used, no chi-square statistic is provided, yet this is needed to compute Cramer’s *V*. To be able to report an effect size for the three variables analysed using Fisher’s exact test, the *N − 1* chi-square test was also performed to obtain a chi-square statistic, in order to obtain an effect-size measure.

2 To interpret the effect sizes, we used guidelines for effect size interpretation as reported by Cohen (1988, 1992) and echoed by Kim (2017). For *V* (*df* = 1) and *r*, 0.10 denotes a small effect, 0.30 a medium effect, and 0.50 a large effect. For *V* (*df* = 2), 0.07 denotes a small effect, 0.21 medium, and 0.35 large. For *V* (*df* = 3), 0.06 denotes a small effect, 0.17 medium, and 0.29 large.
